# Malignant adenomyoepithelioma combined with adenoid cystic carcinoma of the breast: a case report and literature review

**DOI:** 10.1186/1746-1596-9-148

**Published:** 2014-07-23

**Authors:** Ying Yang, Yanmei Wang, Jinsong He, Guoqing Pan, Xiaoyu Tuo, Aimei Jiang, Li Bian

**Affiliations:** Department of Pathology, The First Affiliated Hospital of Kunming Medical University, 295, Xichang road, Kunming, Yunnan China; Department of Breast surgery, The First Affiliated Hospital of Kunming Medical University, Kunming, Yunnan China

**Keywords:** Breast, Adenomyoepithelioma, Adenoid cystic carcinoma

## Abstract

**Background:**

Malignant adenomyoepithelioma and adenoid cystic carcinoma are both rare malignant epithelial-myoepithelial tumors of the breast. We report a very rare case with a malignant adenomyoepithelioma combining with adenoid cystic carcinoma in a single mass.

**Case presentation:**

A 61-year-old female presented with a palpable painless mass in the right breast. Mammography revealed a large irregular dense shadow without obvious internal calcification. A simple lumpectomy was performed, and a 1.6cm well-circumscribed pale-tan nodule was presented. Histologically, the nodule was composed of two obscure lobules. One exhibited typical histological image of adenoid cystic carcinoma, the other one showed the image of epithelial-myoepithelial carcinoma of salivary gland, and support the diagnosis of biphasic malignant adenomyoepithelioma. Transition between the two lobules was gradual. Immunohistochemically, CK18 and P63 highlighted the epithelial and myoepithelial cells respectively in both lesions. CD117 was positive in the epithelial cells of adenoid cystic carcinoma, but was totally negative in malignant adenomyoepithelioma.

**Conclusion:**

This report is, to our knowledge, the first case that combines these two tumors in a single mass. In addition, we present a review of the literature. The histogenesis of these tumors is also discussed.

**Virtual Slides:**

The virtual slide(s) for this article can be found here: http://med.motic.com/MoticGallery/Slide?id=D562817E-23C2-4F72-9823-86EF6DA40005&user=2C69F0D6-A478-4A2B-ABF0-BB36763E8025 and http://med.motic.com/MoticGallery/Slide?id=38BB7126-6FFB-4B66-A208-B8C0F528DCA8&user=2C69F0D6-A478-4A2B-ABF0-BB36763E8025

## Background

Malignant adenomyoepithelioma (AME) and adenoid cystic carcinoma (ACC) are both rare malignant epithelial-myoepithelial lesions of the breast. There are no more than 50 documented cases for the former [1], and the latter constitutes about 0.1% of all breast tumors [2]. Difficulties are commonly encountered in diagnosis and differential diagnosis because of the rarity and similarity of these two tumors. Composed of epithelial and myoepithelial cells, these two salivary gland-like tumors of the breast are morphologically close. However, the relationship of them was seldom observed. There is only one report of adenoid cystic carcinoma arising within an adenomyoepithelioma so far [3].To the best of our knowledge, what we present herein is the first case that combines malignant adenomyoepithelioma and adenoid cystic carcinoma in a single mass. The aim of this report is to analyze the histological and immunohistochemical features of the two tumors by comparing each other, conclude the method of differential diagnosis, and most importantly, apply clue to the genesis of epithelial-myoepithelial tumors.

## Case presentation

**Figure 1:**
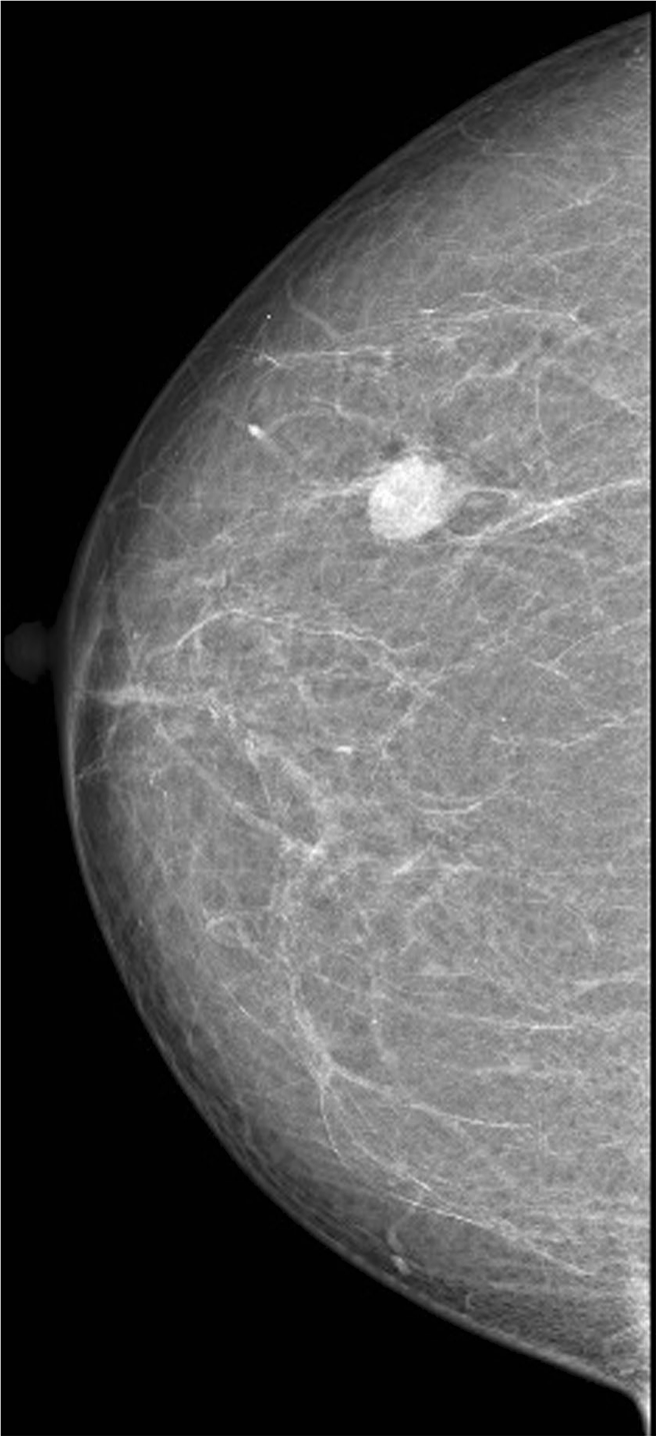
**Imaging inspection.** Mammography revealed a small irregular dense shadow in the upper outer quadrant of the right breast.

**Figure 2:**
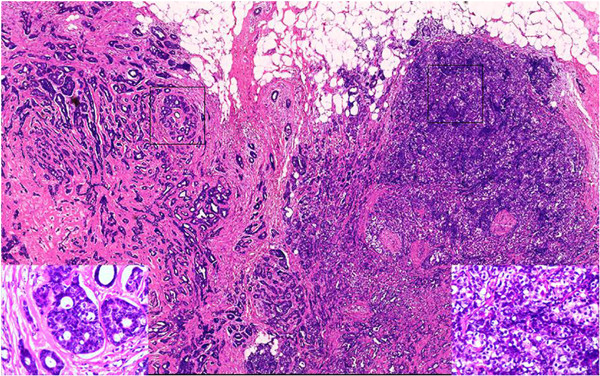
**Low-power examination.** The nodule was composed of two obscure lobules on low-power examination. The left one was adenoid cystic carcinoma and the right one was malignant adenomyoepithelioma. HE, ×10.

**Figure 3:**
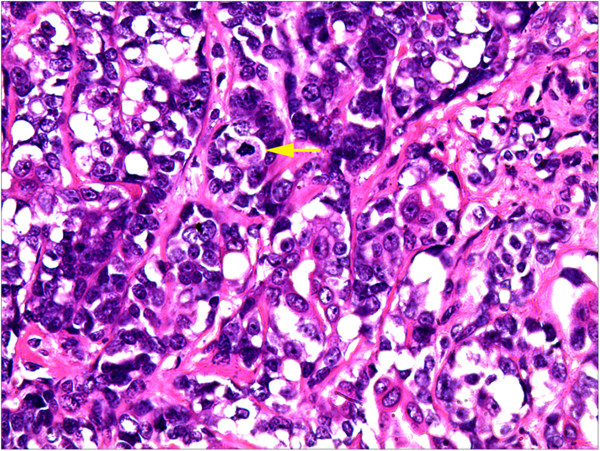
**High-power examination to the malignant adenomyoepithelioma.** Atypia was obvious in both epithelial and myoepithelial cells with moderate degree of nuclear pleomorphism, prominent nucleoli, high nuclear cytoplasmic ratio and increased mitotic figures (arrow). HE, ×200.

**Figure 4:**
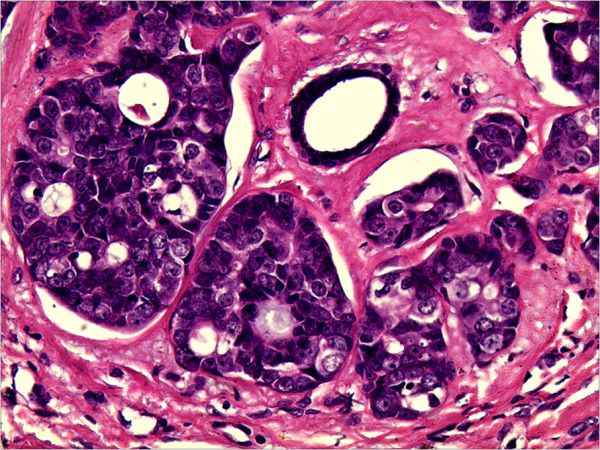
**High-power examination to the adenoid cystic carcinoma.** The cribriform structure in adenoid cystic carcinoma contains true glandular spaces and pseudolumens. HE, ×200.

**Figure 5:**
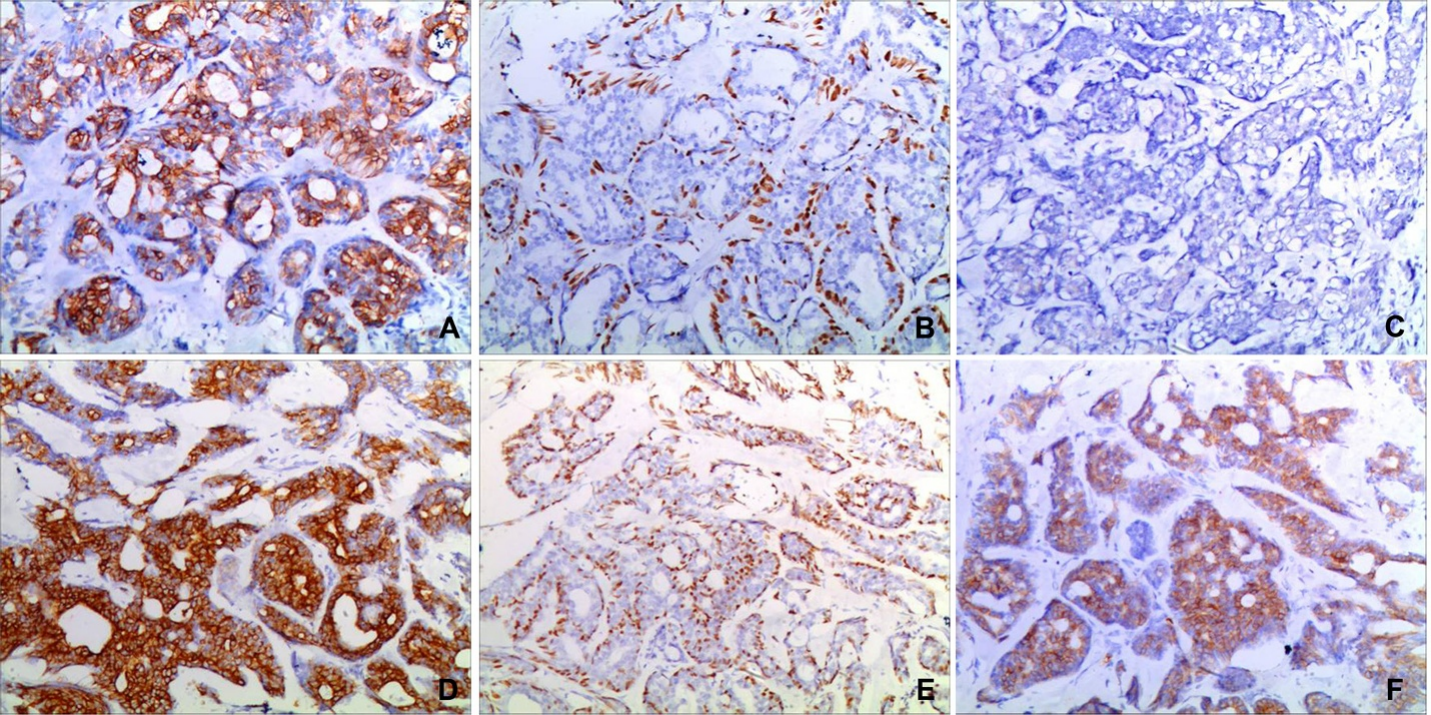
**The immunohistochemistry panel of malignant adenomyoepithelioma (A, B, C) and adenoid cystic carcinoma (D, E, F).** CK18 was expressed in epithelial cells of malignant adenomyoepithelioma **(A)** and adenoid cystic carcinoma **(D)**. P63 was expressed in myoepithelial cells of malignant adenomyoepithelioma **(B)** and adenoid cystic carcinoma **(E)**, CD117 was negative in malignant adenomyoepithelioma **(C)** and positive in adenoid cystic carcinoma **(F)**. ×100.

A 61-year-old female was admitted to The First Affiliated Hospital of Kunming Medical University, Yunnan, China, complaining of a lump in the right breast which had appeared 2 days earlier and had been increasing in tenderness for a week. During the physical examination, the patient’s breasts were found to be bilaterally symmetrical, without any skin retraction. The two nipples were on the same horizontal line without discharge nor retraction. A mass was palpable in the upper-outer quadrant about 5 cm away from the right nipple, with an approximate size of 1.5 cm × 1 cm. The mass was tough in texture, irregular in shape, unclear in boundary and slightly adhesive to the surrounding tissues. There were no positive findings in the left breast. The superficial lymph nodes were not palpable in the bilateral axillary and clavicular fossa. The patient had no history of smoking or alcohol consumption and there was no family history of any types of tumor.Ultrasonography revealed an ill-defined mass in the right breast, and its internal echo was non-homogenous. Mammography revealed a small irregular dense shadow in the upper outer quadrant of the right breast (Figure 1). The boundary of the shadow was unclear and the glands around were gathered without obvious internal calcification. The emission computed tomography (ECT) of whole-body bone imaging and the CT of brain, liver and lung imaging showed no signs of tumor metastasis. Evaluation of tumor markers showed that serum carbohydrate antigen (CA) CA 15–3 was 37.6 U/ml (the normal range is 0–35 U/ml). Other tumor markers were within normal ranges. A simple lumpectomy was performed in the hospital.Grossly, the well-circumscribed pale-tan nodule measured 1.6cm × 1.2cm × 0.5cm and lacked a distinct capsule. Histologically, the nodule was composed of two obscure lobules without evidence of a capsule on low-power examination (Figure 2). One of the lobules consisted of small tubular ducts formed by two phenotypically distinct cell layers. The inner epithelial cells exhibited eosinophilic cytoplasm and the outer myoepithelial cells were clear. Tubular structures predominated in this lobule and formed an expansile circumscribed, partially-encapsulated mass, although focally infiltrated into adjacent fat tissue (Figure 2). A papillary component was also noted in some areas. In addition, atypia was obvious in both epithelial and myoepithelial cells with moderate degree of nuclear pleomorphism, prominent nucleoli, high nuclear cytoplasmic ratio and increased mitotic figures (11/10HPF) (Figure 3). These histological features support the diagnosis of malignant adenomyoepithelioma.The other lobule of the mass exhibited typical histological image of adenoid cystic carcinoma which consisted of tubular and cribriform structure and had infiltrative borders. The epithelial and myoepithelial cells, whose difference was not as obvious as in adenomyoepithelioma, were polarized around two types of spaces: true glandular spaces (contain mucoid material) and pseudolumens (contain basement membrane material) (Figure 4). The myoepithelial cells of adenoid cystic carcinoma were smaller, had a more hyperchromatic and basaloid character and had much less cytoplasm than those of adenomyoepithelioma, and the invagination of stroma was frequently present. Transition from adenomyoepithelioma to adenoid cystic carcinoma was gradual. And we can also see adenomyoepithelioma and adenoid cystic carcinoma combined in some areas.Immunohistochemistry can distinguish the two different cell types. In both lesions, the myoepithelial cells were highlighted by CK5/6, smooth muscle actin (SMA) and p63, while the epithelial cells were positive for CK18 (Figure 5). Immunostains for estrogen (ER) and progesterone (PR) were negative in AME and ACC but positive in surrounding normal ductal epithelial, and human epidermal growth factor receptor 2 (Her2) were also negative in this case. Nevertheless, CD117 expression was found in adenoid cystic carcinoma in contrast to adenomyoepithelioma, where CD117 was not expressed (Figure 5).

A modified radical mastectomy was performed according to the pathology results, the patient’s economic situation and with the patient’s consent. No lymph nodes involvement was found (0/21). The margins, basement membrane and nipple were all free of metastasis. Chemotherapy and radiation therapy were not indicated and the patient remained in a good condition throughout the 12-month follow-up period.

## Discussion

Adenomyoepithelioma is usually a benign neoplasm with a low metastatic potential. However, the malignant transformation can arise from the epithelial component, the myoepithelial component, or both. There are no well-received criteria for malignant adenomyoepithelioma because of the rarity of this lesion. According to the growth pattern of malignant components, the cases reported in the literatures can be divided into two types: 1) an area of obvious malignant component arising in a typical low-grade adenomyoepithelioma, which may be more appropriately named “adenomyoepithelioma with carcinoma” as defined in the 4^th^ edition of WHO Classification of Tumours of the Breast [4], or “carcinoma arising in adenomyoepithelioma”; 2) an adenomyoepithelioma with apparent cytological atypia, increased mitotic activity (>5/10HPFs), invasive growth pattern, and/or presence of necrosis in both epithelial and myoepithelial cells as the histological image of the current case, reminiscent of epithelial-myoepithelial carcinoma of salivary glands [5, 6]. This type was named by Marian et al. as “atypical adenomyoepithelioma” [1] and may be more appropriately named “epithelial-myoepithelial carcinoma of the breast” in our opinion. Base on the differentiation of the malignancy, the tumors can be divided into monophasic and biphasic. The monophasic malignancy could be invasive ductal carcinoma not otherwise specified, metaplastic carcinoma, low-grade adenosquamous carcinoma [5], invasive lobular carcinoma [7], and ductal carcinoma in situ [8, 9] which arising from epithelial component, or malignant myoepithelioma [1] which arising from myoepithelial component. Only 16 biphasic malignant adenomyoepitheliomas in which malignancy arising from both epithelial and myoepithelial cells have been reported previously [5, 6, 10–17] (Table 1), and our current case is another one.

**Table 1:** **Reported cases of biphasic malignant adenomyoepithelioma**

Reference	No. of cases	Age (year)	Size (cm)	Histologic appearance of malignancy	Presence of metastasis
Hayes [5]	3	77 ~ 93	3.2 ~ 14.0	One epithelial-myoepithelial carcinoma with chondrosarcoma	1 lung
				One epithelial-myoepithelial carcinoma	1 lost to follow up
				One epithelial-myoepithelial carcinoma/ACC like	
Ahmed, et al. [6]	1	71	1.0	Nests and cords of both epithelial and myoepithelial tumor cells	None
Qureshi, et al. [10]	1	65	13	Epithelial glandular structures and myoepithelial spindle areas.	None
Hungermann, et al. [11]	1	60	Not reported	Biphasic malignancy with well-developed tubular structures	Not reported
Simpson, et al. [12]	1	50	4.0	Mixed osteogenic, spindle cell, and carcinomatous differentiation	Lung
Kiaer, et al. [13]	1	46	2.0	Peripherally proliferating myoepithelial cells and luminally localized eosinophilic cuboidal and columnar cells.	None
Pauwels, et al. [14]	1	49	Not reported	A proliferation of tubules composed of epithelial cells surrounded by myoepithelial cells with high mitotic figures and focal atypia	None
Petrozza, et al. [15]	1	60	2.0	Focal epithelial-like component with high mitotic figures and partial myoepithelial-like component with atypia and high mitotic figures	None
Rasbridge, et al. [16]	5	39 ~ 76	1.3 ~ 4.5	Contain areas of invasive malignant cells or foci of cellular atypia and increased mitotic activity.	1 brain
Trojani, et al. [17]	1	51	2.0	Bicellular pattern of epithelial and myoepithelial cells	lung
Yang and Wang, et al.(current case)	1	61	1.6	Epithelial-myoepithelial carcinoma	None

ACC: adenoid cystic carcinoma.

Adenoid cystic carcinoma is another neoplasm in the spectrum of hyperplastic and neoplastic lesions characterized by dual differentiation into ductal and myoepithelial cells [4]. There was only one report that adenoid cystic carcinoma arising in an adenomyoepithelioma in breast [3], which Hayes commented that “since these neoplasms are so closely related this is an arguable entity” [5]. However, the difference does exist between the two entities. Adenoid cystic carcinoma has a characteristic cribriform architecture, the formation of true glandular spaces and pseudolumens, and the histologically distinct invagination of stroma. It lacks the papillary architecture which is frequently seen in adenomyoepithelioma. The myoepithelial cells tend to be smaller and more basaloid, and the arrangement of epithelial and myoepithelial cells is less irregular than that of adenomyoepithelioma [5, 18]. Immunohistologically, CD117 highlights the epithelial cells of adenoid cystic carcinoma [4, 19, 20] but is totally negative in malignant adenomyoepithelioma.

The current case and the only case reported previously with an ACC focus next to AME mass have their similarities and differences (Table 2). Interestingly, we recently consulted a case of a 45-year-old female who received a lumpectomy three years ago with a pathological image of adenoid cystic carcinoma. The recurrent tumor showed two components of adenoid cystic carcinoma and malignant adenomyoepithelioma. However, we are not fortunate enough to get the pathological documentation of this case. Tumors with epithelial-myoepithelial differentiation are rare in breast but comprise a wide spectrum of lesions ranging from benign (such as pleomorphic adenoma and adenomyoepithelioma) to low grade malignant (such as malignant adenomyoepithelioma and adenoid cystic carcinoma). Boecker and Buerger have demonstrated the presence of pluripotent progenitor cells which have the potential to differentiate into either glandular or myoepithelial cells [21]. It is possible that adenoid cystic carcinoma and malignant adenomyoepithelioma develop from the same pluripotent progenitor cells [3], so the separation for these two morphologically close lesions could be somewhat arbitrary. They could coexist and even transform to each other under certain conditions.

**Table 2:** **Comparison between the current case and the previous case**

	Sex	Age (year)	Location	Size (cm)	Type of AME	Proportion of AME to ACC	Relationship between AME and ACC
Van Dorpe et al. [3]	Female	36	Juxta-areolar in right breast	1.0	Low-grade AME	1.0: 0.3	A focus of ACC at the edge of AME penetrated the capsule
Yang et al. (current case)	Female	61	Upper-outer quadrant of right breast	1.6	Malignant AME	1:1	ACC as a lobule of the mass combined with malignant AME

ACC: adenoid cystic carcinoma; AME: adenomyoepithelioma.

Though ACC and AME are triple-negative breast cancers,which show a poor prognosis when paired with basal-like transcriptome, hormone receptor negativity status is not related to poor differentiation and a worse prognosis, as defined in invasive ductal carcinoma not otherwise specified (IDC NOS) [22–25]. In addition, compared with adenoid cystic carcinoma of salivary, although they have similar morphological and immunological phenotypes and even the same molecular genetic defect, the t(6;9)(q22–23;p23–24) translocation [26], the biological behavior of breast and salivary ACC is different. The former showed indolent clinical behavior, and the latter showed an aggressive course. Adenoid cystic carcinoma of breast presents as a localized disease with a low frequency of axillary lymph node involvement of less than 8%, and rare distant metastases of fewer than 20% [27–30]. Therefore, most clinicians recommend a breast-conserving surgical therapy with or without radiotherapy [31]. However, some research performed grading according to the criteria accepted for salivary ACC and suggested that grade 3 (solid growth pattern) may have a higher propensity of recurrence and metastasis [4]. This result has not been confirmed by long-term follow-up data. At the same time, AME of breast has better prognosis comparing with epithelial-myoepithelial carcinoma of salivary gland [32].Most of the adenomyoepitheliomas of breast behave as benign though rare cases have metastasized [33]. The behavior of malignant adenomyoepithelioma seemed to be related to the grade of the malignant component and the tumor size [4, 5]. In our case, the component of high grade epithelial-myoepithelial carcinoma might indicate a high frequency of recurrence and metastases. To date, there are limited published data on the biological behavior and long-term clinical outcome of adenoid cystic carcinoma combining with malignant adenomyoepithelioma. As a result, it is necessary for the patient to be closely followed-up and periodically examined following treatment.

## Conclusion

In this paper, we report the case of a rare biphasic malignant adenomyoepithelioma combining with adenoid cystic carcinoma in a single mass of a 61-year-old female. The histological characters of the two tumors were typical, and the transition from malignant AME to ACC was gradual. Immunohistochemistry was helpful in distinguishing these two types of malignant epithelial-myoepithelial tumors. Although most ACC has a more favorable clinical outcome, the component of high grade epithelial-myoepithelial carcinoma in this case might indicate a high frequency of recurrence and metastases.

## Consent

Written informed consent was obtained from the patient and her family for publication of this Case Report and any accompanying images. A copy of the written consent is available for review by the Editor-in-Chief of this journal.

## Abbreviations

AME: Adenomyoepithelioma
ACC: Adenoid cystic carcinoma
ECT: Emission computed tomography
CA: Carbohydrate antigen
SMA: Smooth muscle actin
ER: Estrogen
PR: Progesterone
Her2: Human epidermal growth factor receptor 2.
